# New insights on diagnosis and treatment of AVP deficiency

**DOI:** 10.1007/s11154-023-09862-w

**Published:** 2023-12-13

**Authors:** Julie Refardt, Cihan Atila, Mirjam Christ-Crain

**Affiliations:** 1 Departments of Endocrinology, Diabetology and Metabolism University Hospital Basel, Basel, Switzerland; 2 University of Basel, Basel, Switzerland; 3 Department of Internal Medicine, Section of Endocrinology, Erasmus Medical Center, Rotterdam, The Netherlands

**Keywords:** Copeptin, Diabetes insipidus, Primary polydipsia, Hypertonic saline stimulation test, Arginine stimulation test, Glucagon stimulation test, Polyuria polydipsia syndrome, Arginine vasopressin

## Abstract

Arginine vasopressin deficiency (AVP-D) is one of the main entities of the polyuria-polydipsia syndrome. Its correct diagnosis and differentiation from the other two causes - AVP resistance and primary polydipsia – is crucial as this determines the further management of these patients.

Over the last years, several new diagnostic tests using copeptin, the stable surrogate marker of AVP, have been introduced. Among them, hypertonic saline stimulated copeptin was confirmed to reliably and safely improve the diagnostic accuracy to diagnose AVP-D. Due to its simplicity, arginine stimulated copeptin was put forward as alternative test procedure. Glucagon-stimulated copeptin also showed promising results, while the oral growth hormone secretagogue Macimorelin failed to provide a sufficient stimulus. Interestingly, an approach using machine learning techniques also showed promising results concerning diagnostic accuracy.

Once AVP-D is diagnosed, further workup is needed to evaluate its etiology. This will partly define the further treatment and management. In general, treatment of AVP-D focuses on desmopressin substitution, with oral formulations currently showing the best tolerance and safety profile. However, in addition to desmopressin substitution, recent data also showed that psychopathological factors play an important role in managing AVP-D patients.

## Introduction

**Fig. 1:**
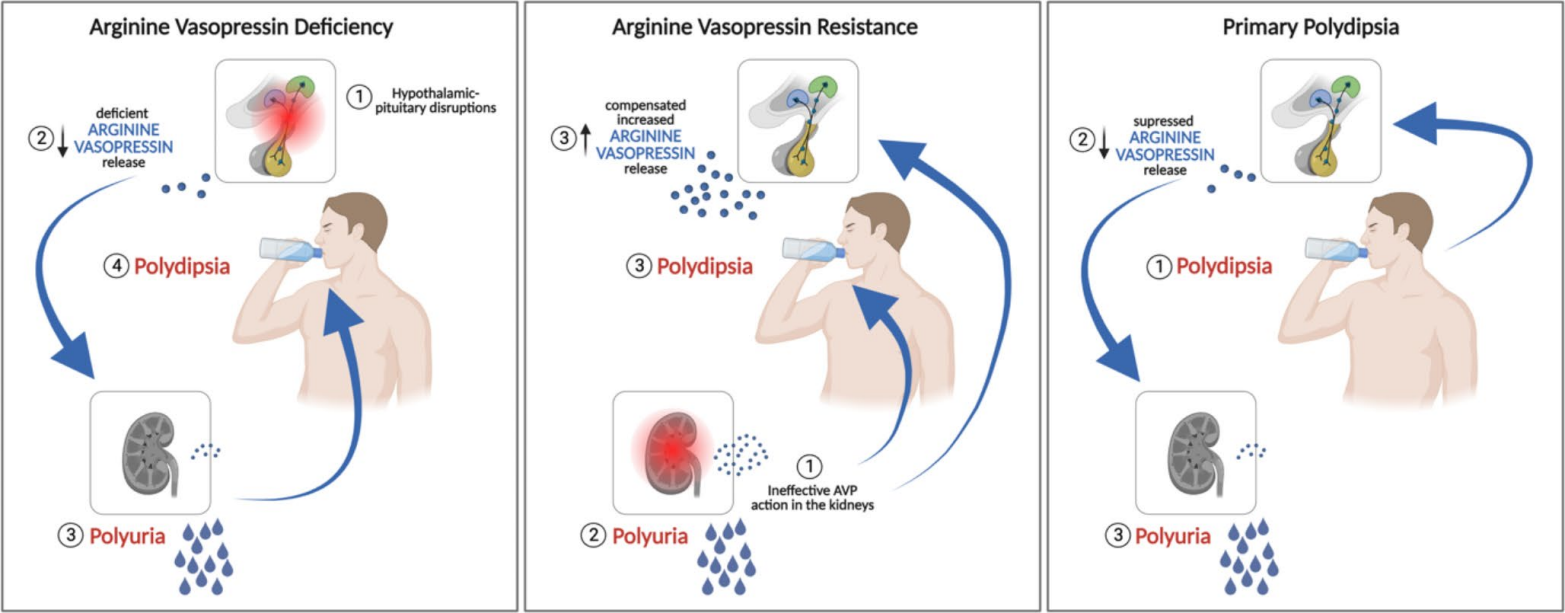
Schematic overview over the main disorders behind polyuria polydipsia syndrome

Arginine vasopressin deficiency (AVP-D, formerly known as central diabetes insipidus) is the main disorder developing from disruption of the hypothalamic-posterior pituitary axis and has been challenging endocrinologists, nephrologists and internal medicine specialists alike. AVP-D is one of the main entities of the polyuria-polydipsia syndrome – which is characterized by a high output of hypotonic urine accompanied by elevated fluid intake [1]. The two other main etiologies of this disorder are AVP-R (AVP-R, formerly known as nephrogenic diabetes insipidus), characterized by renal insensitivity to AVP and primary polydipsia (PP), where affected patients have elevated fluid intake despite initial adequate AVP secretion and renal response to it (Fig. 1).

There are many different disorders that can lead to polyuria-polydipsia syndrome, as outlined in Table 1. This emphasizes the importance of finding the correct diagnosis and following management.

**Table 1:** Etiologies of polyuria polydipsia syndrome

AVP Deficiency		
Trauma		Pituitary surgery Deceleration injury Radiotherapy
Primary tumors		Craniopharyngioma Meningioma Germinoma Rathke’s cleft cyst Pituitary adenoma Astrocytoma
Metastatic cancer		Lymphoma Breast cancer Lung cancer
Infiltrative		Neurosarcoidosis Langerhans cell histiocytosis
Inflammatory / autoimmune		Lymphocytic hypophysitis Granulomatous hypophysitis Xanthomatous hypophysitis IgG4 related hypophysitis
Infectious		Meningitis Encephalitis Tuberculosis
Idiopathic		
Hereditary		Mainly affected gene: *AVP*
AVP Resistance		
Drugs		Lithium Cisplatin Demeclocycline
Electrolyte disorders		Hypokalaemia Hypercalcemia
Haematological		Multiple Myeloma Amyloidosis Sickle cell disease
Hereditary		Mainly affected genes: *AVPR2, AQP2*
Gestational AVP Deficiency		
Increased AVP degradation		by placental vasopressinase
Primary polydipsia		
Psychogenic Habitual Somatic, e.g. hypothalamic lesions Beer potomania		

With the discovery of copeptin [2, 3], the stable surrogate marker of AVP, the diagnostic evaluation of AVP-D greatly improved. Over the last years, several copeptin-based tests showed their superiority to the standard water deprivation test [4–6].

Concerning the management of AVP-D, current data revealed the importance of a holistic evaluation of AVP-D patients. In addition, new data emerged concerning the optimal treatment.

This overview will first discuss the different entities and causes of polyuria-polydipsia syndrome as well as the role of copeptin in its evaluation in adult patients. It will then focus on the different available diagnostic tests and explain their procedures. Lastly, the treatment and management of AVP-D patients, including the importance of evaluating psychopathological factors, will be discussed.

## Background

### Polyuria polydipsia syndrome

The polyuria-polydipsia syndrome is characterized by a high urinary output of more than 50 ml per kg body weight per 24 h, accompanied by polydipsia of more than 3 L a day [1]. After the exclusion of AVP-independent causes (such as uncontrolled diabetes mellitus), the differential diagnosis of polyuria-polydipsia syndrome involves the distinction between AVP-D, AVP-R and primary polydipsia (Fig. 1).

In the last years, patient reports were published pointing to confusion of the formerly known disease name diabetes insipidus with the more common diabetes mellitus, resulting in adverse outcomes including death [7]. Likewise, a recent web-based patient survey in more than 1000 patients with AVP-D demonstrated that more than 80% of patients had experienced confusion of their condition with diabetes mellitus by health-care professionals on at least one occasion. Moreover, study participants indicated that this insufficient understanding of their disease affected the management of their condition. Importantly, 85% of participants preferred a renaming of the condition, with the clear wish to not use the term diabetes in the name of the disease [8]. Therefore, a working group including members of the main endocrinology societies worldwide was assambled to discuss and propose alternative names. According to the common propositions, the name of central diabetes insipidus was changed to AVP-D, and nephrogenic diabetes insipidus was changed to AVP-R [9]. Accordingly, this new nomenclature is now used throughout this article.

AVP-D results from inadequate secretion and usually deficient synthesis of AVP in the hypothalamic neurohypophyseal system in response to osmotic stimulation. Mostly, thirst mechanisms are intact, leading to compensatory polydipsia. However, in a variant of AVP-D called osmoreceptor dysfunction, thirst perception is also impaired, which can result in serious complications associated with hyperosmolality. AVP-D is mostly acquired due to disorders that disrupt the neurohypophysis, including pituitary surgery, tumors, trauma, hemorrhage, thrombosis, infarction, or granulomatous disease [1] (Table 1*)*. Less commonly, it is congenital by genetic mutations of the AVP gene [10].

In contrast, AVP-R is most commonly congenital due to mutations in the gene for the AVP V2R (X-linked recessive pattern of inheritance) or in the gene for the AQP2 water channel (autosomal recessive pattern of inheritance) [11]. It can also be acquired by drugs, most prominently lithium, but also other drugs interfering with urine concentration, or it can be due to electrolyte disorders, i.e., hypercalcemia or hypokalemia. For both forms, AVP-D and AVP-R, complete and partial defects have been described [12], further increasing the difficulty in their distinction.

Lastly, primary polydipsia is characterized by an excessive fluid intake leading to polyuria even in the presence of intact, although suppressed, AVP secretion and appropriate renal response. It is mostly seen in patients with psychiatric disease, in which case it is also called psychogenic polydipsia. It may, however, also be caused by a defect in the thirst mechanism (called dipsogenic diabetes insipidus) or occur habitually.

### Copeptin

AVP comprises 9 amino acids and is synthesized as part of the 164 amino acid precursor protein peptide pre-pro-Vasopressin in magnocellular neurons located in two discrete areas of the hypothalamus, the supraoptic and paraventricular nucleus. The precursor peptide comprises the AVP moiety, neurophysin-2, and a 39 amino acid long glycosylated peptide with a leucine-rich core segment, termed copeptin [2, 3]. Two neuroendocrine mechanisms are involved in the production and release of AVP. In the first one, exocytotic release is determined mainly by the effective osmotic pressure of the extracellular body fluid and by more pronounced decreases in extracellular volume. A second AVP neurosecretory pathway transports high concentrations of the hormone from parvocellular neurons to the pituitary portal system, where AVP acts synergistically with corticotropin-releasing hormone to stimulate adrenocorticotropic hormone (ACTH) release from the anterior pituitary [13, 14]. Consequently, the main physiologic function of AVP is the homeostasis of fluid balance, vascular tonus and regulation of the endocrine stress response.

Much less is known about the physiological function of copeptin. It was first postulated that copeptin has a role as a prolactin-releasing factor, however with inconclusive results [15, 16]. More recent data suggest copeptin to be a chaperone-like molecule which is involved in the structural formation of the precursor hormone [17]. The tight regulation of copeptin in the circulation suggests that copeptin has a specific peripheral function, although experimental data so far prove no evidence for this. Copeptin responds as rapidly as AVP to osmotic, hemodynamic, and unspecific stress-related stimuli, which is explained by its equimolar production together with AVP. Copeptin is rapidly eliminated. It appears that copeptin is processed either by tissue-bound proteases and/or is rapidly eliminated via the kidneys or the liver. Even though no specific copeptin receptor or copeptin elimination mechanisms are known today, the fact that copeptin can be measured in the kidneys indicates at least partly elimination via the kidneys.

AVP is difficult to measure and therefore was not implemented in clinical routine due to complex pre-analytical requirements, the lack of readily available and fast assays and the fact that a large amount of AVP in the circulation is bound to platelets, resulting in underestimation of actual AVP concentration, whilst incomplete removal of platelets from plasma or prolonged storage of unprocessed blood leads to falsely elevated AVP measurements [18–20]. In addition, detection of AVP is also hampered by its short in vivo half-life of less than 30 min [21] and its instability in isolated plasma, even when stored at − 20 °C.

Due to its high ex vivo stability and simple and robust measurement, copeptin offers a simple alternative method to assess the release of AVP indirectly. There are several copeptin assays available. The two assays with sufficient technical description and clinical data justifying their routine clinical use are the original sandwich immunoluminometric assay (LIA) [22] and its automated immunofluorescent successor (on the KRYPTOR platform). A direct comparison between copeptin and AVP release in relationship to serum osmolality using the well-established AVP assay showed a stronger correlation for serum osmolality with copeptin than with AVP and a very strong correlation between both peptides [23]. A study directly comparing the decay kinetics and half-life of copeptin and AVP again showed very similar kinetics of copeptin in response to changes in osmotic pressure to AVP. The half-life of copeptin was around 2 times higher than the half-life of AVP, reflecting the differing volume distribution and metabolic clearance rates of the two peptides [24].

The normal range for copeptin under normo-osmotic baseline conditions showed a median copeptin plasma concentration of 4.2 pmol/L with a broad range between 1 and 13.8 pmol/L^22^. Similar concentrations were found in a random population of 5000 individuals [25]. Men consistently showed slightly higher values than women, with a difference in median values of about 1 pmol/L. Interestingly however, this gender-specific difference could not be detected in the hyperosmolar range [24]. Copeptin levels show no correlation with age [22], and no circadian variability [26, 27]. Copeptin release seems not to be affected by food intake [28] or the menstrual cycle of women [29], suggesting that copeptin measurements are quite robust and can be reliably interpreted independently of time point of withdrawal, prandial status or menstrual cycle. In contrast, already small amounts of oral fluid intake may significantly decrease copeptin levels, which is important to notice for data interpretation [28]. As mentioned above, copeptin shows the same responsive behavior to osmotic and hemodynamic changes and unspecific stress as demonstrated for AVP.

It is therefore not surprising that copeptin was put forward for the differential diagnosis of polyuria-polydipsia syndrome.

## Diagnosing AVP-D

### Use of clinical, laboratory and radiological data

Since polyuria and polydipsia is the main clinical symptom in AVP-D, AVP-R and primary polydipsia, they are not helpful for their discrimination. Especially partial AVP-D and primary polydipsia showed a great overlap in the amount of polyuria and polydipsia [4]. The same was true for baseline laboratory values such as plasma sodium and osmolality. Other symptoms, such as nocturia or sudden symptoms onset, are viewed to be more typical for AVP-D patients, however depending on the underlying etiology, a slow onset is also possible, e.g. after irradiation or in familial forms (Table 1*).* Psychiatric disorders have been described to be more prominent in patients with primary polydispia [30], however recent prospective data showed a similar rate of around 30% for AVP-D and primary polydipsia patients alike [4]. The presence of psychopathological findings in AVP-D patients will be discussed further in the chapter ‘Treament and Management’.

Magnetic resonance imaging (MRI) is often used to further evaluate patients with suspected AVP-D. Here, the absence of an area of hyperintensity in the posterior pituitary, the so-called bright spot, was long thought to be pathognomic for AVP-D since it is believed to result from AVP stored in neurosecretory granules [31, 32]. However, an age-related absence of the bright spot was later shown in healthy volunteers [33]. In addition, its absence was shown in 70% of patients with AVP-D but also 39% of patients with primary polydipsia in a prospective study involving 92 patients with polyuria-polydipsia syndrome [4], meanwhile several reports observed a persistent bright spot in AVP-D patients [34, 35]. The second typical MRI characteristic, a thickened pituitary stalk, is also not specific for AVP-D [4, 36]. However, discovering these findings should lead to a thorough evaluation for pituitary or hypothalamic disorders [37] (Table 1).

However, the combination of clinical and MRI information can be very useful to evaluate patients with suspected AVP-D. This was recently confirmed in a study using machine learning techniques [38]. Using the laboratory parameters urine osmolality, plasma sodium and glucose as well as the clinical information on whether patients had transsphenoidal surgery or known anterior pituitary deficiencies, the machine learning-based algorithm resulted in a high area under the curve score of 0.87 to diagnose AVP-D. This score was further improved to 0.93 by adding the MRI parameter pituitary stalk enlargement. However, until machine learning-based algorithms become more standardized, diagnostic tests for AVP-D are needed.

### Diagnostic tests

#### Indirect water deprivation test

Described in 1970, the indirect water deprivation test was the diagnostic gold standard for decades [12]. Its name derives from the indirect diagnostic assessment using the urinary concentration ability over a fluid restriction period of 16 h as well as its change to the administration of the synthetic AVP variant desmopressin at the end of the test. Patients were urinary osmolality stays below 300 mosm/kg but increases over 50% upon desmopressin administration are diagnosed as having complete AVP-D. Meanwhile patients with a persistent low urinary osmolality and no reaction to desmopressin are diagnosed with AVP-R. Urinary concentration in patients with partial AVP and primary polydipsia is expected to increase to 300–800 mosm/kg, with a further increase in osmolality upon desmopressin injection of more than 9% in partial AVP-D patients and less than 9% in patients with primary polydipsia.

Although this evaluation feels intuitive and an overnight water deprivation test is often done to evaluate the severity of symptoms, it is important to note that these cutoffs were derived from a single study involving only 36 patients with post-hoc assessment that has never been prospectively validated [12, 39]. Furthermore, the test results can be misleading in primary polydipsia patients with a reduced renal medullary concentration gradient or partial AVP-R patients with a sensitive response to desmopressin administration. In view of these limitations, it was not surprising that two prospective studies evaluating the diagnostic accuracy of the indirect water deprivation test found the diagnostic accuracy to be only 70–77%, with especially low accuracy in the difficult differentiation between partial AVP-D and PP patients [4, 40].

#### Copeptin based tests

Around the same time when the indirect water deprivation test was established, also a direct measurement of AVP upon osmotic stimulation was described. While AVP levels of patients with AVP-D were below a calculated area of normality, those of AVP-R patients were elevated and those of patients with primary polydipsia were within [41]. However, direct AVP measurement has not found its way into clinical practice due to the technical limitations of the AVP assay described above [22, 42, 43] as well as the low diagnostic accuracy of commercially available AVP assays [39, 40]. But with the discovery of copeptin as a reliable AVP surrogate marker [24], the direct approach was re-evaluated.

A first improvement of the diagnostic evaluation was the observation from two prospective studies that patients with AVP-R have elevated baseline copeptin levels ≥ 21.4 pmol/L, without any prior water deprivation [40, 44]. With a sensitivity and specificity of 100%^44^, no further stimulation tests are needed in these patients.

Unfortunately, basal copeptin levels significantly overlap in patients with AVP-D and primary polydipsia [4, 44]. Therefore, to reliably diagnose AVP-D, a stimulation test for copeptin is required. Unfortunately, data evaluating copeptin measurement after the water deprivation test showed a low diagnostic accuracy [4], accordingly stronger stimulators are needed.

In the following, the currently available test methods are described.

#### Hypertonic saline stimulation test

**Fig. 2:**
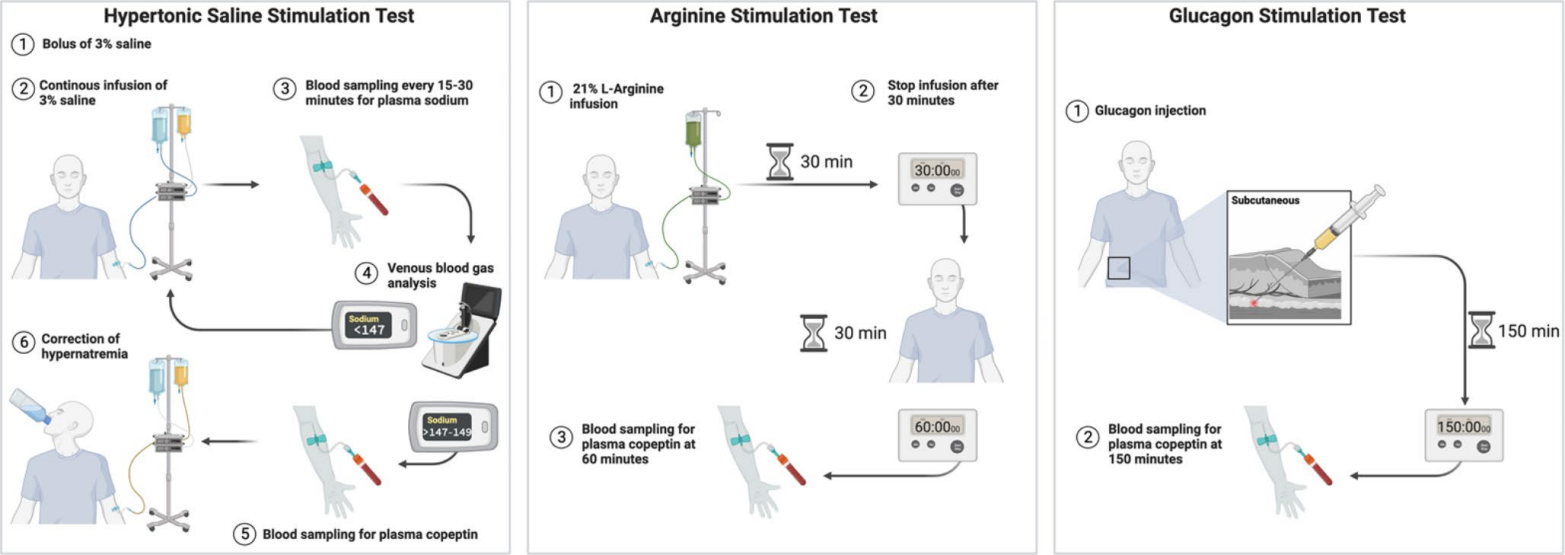
Schematic overview of the copeptin based diagnostic tests for polyuria polydipsia syndrome

Osmotic stimulation, i.e., increasing plasma sodium levels, is a strong trigger for the secretion of AVP and, thus copeptin. That osmotically stimulated copeptin levels can reliably distinguish between patients with AVP-D and primary polydipsia was first shown in a study involving 55 patients [44] and then later confirmed in the so far largest prospective study including 156 patients with AVP-D or primary polydipsia [4]. In the confirmation study [4], patients received a bolus of 250 ml of hypertonic (3%) saline followed by an infusion adjusted to body weight (0.15 ml per kg body weight per minute). The aim was to increase plasma sodium levels to ≥ 149 mmol/L, at which time copeptin was measured (Fig. 2). Importantly, sodium levels have to be monitored every 30 min. Using the pre-defined copeptin cut-off of ≤ 4.9 mmol/L, patients with AVP-D were reliably distinguished from patients with primary polydipsia with a diagnostic accuracy of 97%. Adverse events during the test were mainly limited to side effects of the hypernatremia and involved strong thirst, headache and nausea. They completely regressed as soon as plasma sodium levels were re-lowered by oral and parenteral rehydration. However, it is important to note that the correct performance of the hypertonic saline stimulation test is critical to its safety. Clinicians performing the test must have access to rapid sodium measurements (e.g. via venous blood gas analysis) and closely monitor the rise in sodium levels to avoid osmotic overstimulation.

#### Arginine stimulation test

Arginine infusion, known as a stimulator of the anterior pituitary [45, 46], is used as a standard test in the evaluation of suspected growth hormone deficiency, mainly in children [47–49]. However, in a prospective study including healthy volunteers, it was shown that arginine infusion also stimulates the posterior pituitary leading to an increase in copeptin [5]. In the same study, 96 patients with polyuria-polydipsia syndrome were enrolled. After receiving a body weight-adapted (0.5 g per kg body weight) infusion of L-Arginine Hydrochloride (21%) diluted in 500 ml normal saline (NaCl 0.9%) over 30 min (Fig. 2), copeptin was measured at different time points. It was shown that copeptin measured 60 min after the start of the infusion using the cut-off level 3.8 pmol/L had the highest diagnostic accuracy of 93% to differentiate between patients with AVP-D and primary polydipsia [5]. The most common adverse effect was mild nausea which occurred in 48% of the patients. Two patients were excluded from the main analysis due to vomiting, as severe nausea and vomiting are strong non-osmotic AVP/copeptin stimuli [50, 51]. If severe nausea or vomiting occurs during arginine infusion, test results should therefore be interpreted cautiously and can only be used if copeptin concentrations remain low. In all other cases, a confirmation test is recommended.

Sixty patients participated in both the hypertonic saline [4] and the arginine [5] stimulation study. A post-hoc head-to-head comparison of these patients revealed a diagnostic accuracy of 100% for hypertonic stimulated copeptin compared to 93% for arginine stimulated copeptin. Likely, hypertonic saline infusion provides a stronger copeptin stimulus. Meanwhile, its need for constant supervision and availability of rapid sodium measurement is a disadvantage. In the post-hoc comparison, Arginine infusion had a better test tolerance and it has a simpler test protocol. Recently a prospective multicenter trial aiming at validating the derived arginine-stimulated copeptin levels and directly comparing them to hypertonic saline-stimulated copeptin levels in patients with polyuria-polydipsia syndrome was concluded (NCT03572166). The results are expected by the end of 2023.

#### Macimorelin stimulation test

In view of the effect of arginine stimulation on copeptin levels, an oral stimulation test using macimorelin – an oral ghrelin agonist - was evaluated in a diagnostic study involving 28 healthy participants [52]. Unfortunately, while the effect of macimorelin on growth hormone levels was confirmed, no effect was seen on copeptin levels. Accordingly, the macimorelin stimulation cannot be recommended for the diagnostic evaluation of AVP-D patients.

#### Glucagon stimulation test

Another growth hormone stimulator is glucagon. In a recently published diagnostic study including 22 healthy participants, 10 patients with AVP-D and 10 patients with primary polydipsia, its effect on copeptin levels was evaluated [6]. After subcutaneous injection of 1 mg glucagon, copeptin levels were measured at different time points and compared to placebo injection. Indeed, glucagon injection induced copeptin secretion in healthy participants and primary polydipsia patients. Meanwhile, no relevant increase was seen in the AVP-D patients. A copeptin cut-off level of 4.6 pmol/L measured 150 min after glucagon injection had a sensitivity of 100% and a specificity of 90% to discriminate between AVP-D and primary polydipsia patients (Fig. 2). Accordingly, glucagon stimulation could be a novel simple diagnostic test for AVP-D, however the derived copeptin cut-off should be confirmed in a larger cohort, especially including more patients with partial forms of AVP-D.

In summary, of the copeptin stimulation tests currently available, hypertonic saline stimulation [53] provided the highest diagnostic accuracy, while arginine stimulation [5] has a simpler test protocol and better test tolerance. Glucagon sitmulation [6] also provides an attractive alternative test, but has to be validated in a larger cohort.

## Management and complications of AVP-D

### Pharmacological therapy

The main treatment goals in patients with AVP-D are the correction of pre-existing water deficits and the reduction of excessive urinary water loss. In most patients, osmoregulated thirst perception is intact and adequate fluid intake compensates for urinary water loss [54, 55]. Desmopressin, a synthetic AVP analogue and selective V2 receptor agonist, is the current standard of care and is usually initiated after the diagnosis of AVP-D is confirmed [56, 57]. Desmopressin differs from AVP by two amino acids and is available in parenteral, oral and nasal formulations, providing effective long-term control of polyuria. Amino acid modifications prolong the half-life and eradicate the vasopressor potential [58]. Owing to high variability between individuals in bioavailability, the optimal dose, dosing intervals and duration of effectiveness should be determined individually for each patient. The parenteral route is usually only given to inpatients, e.g. perioperatively in the management of transient polyuria or if the oral / nasal applications cannot be given due to any reasons [59, 60]. In oral formulation, peak antidiuretic effect correlates with the zenith of plasma concentration within 2 h of ingestion, which however can be substantially reduced when ingested together with food [61, 62]. The antidiuretic effect of the intranasal application is more variable than the oral formulation and may be reduced by nasal mucosa inflammation, congestion, or scarring [63]. The initial treatment aim is to reduce nocturia, and therefore, the first desmopressin dose is usually given at bedtime, and if needed, a daytime dose is added. In this initial period, patients should be instructed to avoid excessive fluid intake and be educated about hyponatremia symptoms like nausea, vomiting, headache or lethargy. The first days of treatment should be followed by monitoring of plasma sodium to avoid hyponatremia. Once a stable dose of desmopressin is established, annual monitoring of plasma sodium and kidney function should be performed.

Desmopressin treatment provides immediate symptomatic relief; however, the main complication is dilutional hyponatremia and its associated risk of cerebral edema, seizure, coma and even death [64]. Physiologically, fluid intake suppresses AVP secretion, allowing an aquaresis to prevent water retention [65]. Under the effect of desmopressin, however, even modest fluid intake is retained as there is constant antidiuresis until the effects subside, and therefore, dilutional hyponatremia is a common side effect [66]. A high prevalence of hyponatremia of up to 30% was reported in the outpatient setting and might be explained by the lack of education on the correct use of desmopressin [67, 68]. To reduce the risk of hyponatremia, some physicians recommend either:

1. Delaying a dose of desmopressin up to several times per week until aquaresis and breakthrough symptoms, i.e., strong thirst, full bladder, pale urine, and frequent urination occur or.
2. Omitting a desmopressin dose once or several times per week independent of breakthrough symptoms.

This method has been referred to as ‘desmopressin escape’, and the effectiveness of this approach was recently supported by our large survey-based study [68]. The hyponatremia prevalence was 17% in patients performing ‘desmopressin escape,’ 32% in those unaware of ‘desmopressin escape,’ and 26% in patients aware of ‘desmopressin escape’ but not using this method. Patients performing ‘desmopressin escape’ had a significantly lower hyponatremia prevalence compared to those not being aware of this method and to those aware of ‘desmopressin escape’ but not using this method. Endocrinologists should educate patients about these strategies and apply an individualised approach at desmopressin initiation. Additionally, a lower risk of hyponatremia has been suggested with oral desmopressin compared to the intranasal formulation [69, 70]. More precisely, one study reported a 60% risk reduction for hyponatremia in patients who switched from nasal to oral preparation [70]. In addition, post-marketing safety data indicated a lower risk of hyponatremia in oral compared to nasal desmopressin [71, 72]. However, conversely to these data, another study reported a 33% hyponatremia rate within a four-week dose titration period after switching from a nasal to an oral formulation [73]. Although data from our large survey study also do not support the hypothesis on differences between available formulations and hyponatremia prevalence, one should emphasize the high patient-reported rates of switching from nasal to oral formulations, mostly due to patient preference, pointing to overall better symptom control. This clear preference was also reported by Oiso and colleagues in an observational cohort of almost 200 patients demonstrating a 100% preference for oral rather than nasal formulation [56]. However, further prospective data are needed to investigate this in more detail. Switching between preparations may be particularly useful in patients with poor symptom control and fluctuations in the effectiveness of desmopressin, as well as in those prone to frequent hyponatremia.

Even without desmopressin, patients with a functioning osmoregulated thirst perception and free access to water can compensate for urinary water loss through increased fluid intake. Therefore, hypernatremia, an indicator of inadequate fluid balance, rarely occurs in AVP-D patients with free access to fluids [67]. Importantly, limited access to fluids or excessive fluid loss, for example, by non-availability or restricted intake, vomiting or diarrheal illnesses, unconsciousness, or acute concurrent illness, can lead to life-threatening dehydration [67]. In support of this, Behan and colleagues reported concerningly high rates of hypernatremia of around 20%, particularly in in-hospital settings, presumably owing to a lack of knowledge on correct fluid management and treatment failures by the medical team [67]. Importantly, evidence from our cohort study and others reinforces the need to raise awareness and educate medical personnel about correct fluid management and demand the inclusion of desmopressin as a high-alert medication with 24-hour access in hospitals [74, 75]. More precisely, our data indicated that one in four hospitalized patients reported symptoms of dehydration as a result of an inability to use their desmopressin while in a fasting state without intravenous fluid replacement. Such scenarios have been reported in several cases with serious adverse outcomes, including death [74, 76]. If dehydration occurs, the total body water deficit can be estimated using the following formula: 0.6 × premorbid weight × (1 − 140 / [measured Na^+^ in mmol/L]). It is recommended to replace ~ 50% of the calculated free water deficit over the first 24 h of treatment [75, 77]. Patients should be treated with hypotonic fluids enterally or, if required, preferably with 5% dextrose administered intravenously [75].

A particularly vulnerable group of patients are those with osmoreceptor dysfunction. These patients are prone to dehydration and, at the same time, hyponatremia. Treatment involves close sodium monitoring and educating patients to monitor their own hydration status. Generally, patients are instructed to fix daily fluid intake, which can be adjusted in response to changes in body weight, combined with a fixed dose of desmopressin. In this particular group, patients and family members should be advised to seek early medical attention in situations of increased risk of hypo- or hypernatremia.

### Psychopathological changes

Over the past decades, only a few studies investigated psychological comorbidities in patients with AVP-D. Data from smaller studies have indicated that these patients often experience psychological comorbidities or difficulties such as heightened anxiety, depressed mood, alexithymia, sleep disturbances and reduced sexual drives as well as lower quality of life, despite adequate desmopressin therapy [78–80]. Furthermore, difficulties in categorizing emotions through face processing, personality changes, and increased psycho-social comorbidities, including anxiety, depression, and social withdrawal, have been observed [78, 79, 81–85]. These symptoms have been observed in patients with or without additional anterior pituitary dysfunction, which challenges the notion presented in existing data that anterior pituitary hormone dysfunctions are the primary cause of psychological changes and reduced quality of life in these patients. Consistent with these results, our survey data confirmed the high prevalence of self-reported psychological problems (36%) and overall reduced quality of life (64%), which was subjectively related to the onset of the disease and remained present despite treatment [68]. These observations have diagnostic significance, as psychological comorbidities are often used as a hallmark pointing towards primary polydipsia in routine clinical practice, which is therefore also sometimes referred to as ‘psychogenic’ polydipsia [39, 86]. Accordingly, in previous diagnostic algorithms, a history of psychiatric disease in the evaluation of polyuria-polydipsia was put forward as suggestive for primary polydipsia [39]. Interestingly, our recent questionnaire-based evaluation demonstrated comparable high levels of anxiety, alexithymia, and depression, as well as reduced mental health scores in both patients with primary polydipsia and those with AVP-D (submitted for publication). These findings are important in order to increase awareness to not prematurely diagnose primary polydipsia in patients with polyuria and polydipsia with concomitant psychiatric symptoms contrary to the recommendation of previous diagnostic algorithms [39]. In addition, these data highlight the need to sensitize treating physicians to inquire about psychological disorders and difficulties and, if necessary, refer them for further diagnostic and therapeutic assessment.

## Summary

AVP-D is a challenging disorder to diagnose and treat. The stable AVP surrogate marker copeptin significantly improved diagnostic assessment. While hypertonic saline-stimulated copeptin is the best-researched and most accurate test currently available, arginine- and glucagon-stimulated copeptin are easy-to-perform alternative tests.

Once AVP-D is diagnosed, desmopressin is the standard treatment, with current data suggesting that the oral formulation is safer and preferred by patients. Educating patients about the risk of hyponatremia is important, and this risk could be reduced by regularly performing ‘desmopressin escape’. In the meantime, educating healthcare teams about AVP-D and the risk of hypernatremia in hospitalized patients is critical. Hopefully, by changing the name to AVP-D, a first step has been taken to improve patient safety and raise awareness.

## Data Availability

N/A.
